# Risk and consequences of chemotherapy-induced thrombocytopenia in US clinical practice

**DOI:** 10.1186/s12885-019-5354-5

**Published:** 2019-02-14

**Authors:** Derek Weycker, Mark Hatfield, Aaron Grossman, Ahuva Hanau, Alex Lonshteyn, Anjali Sharma, David Chandler

**Affiliations:** 1 Policy Analysis Inc. (PAI), Four Davis Court, Brookline, MA 02445 USA; 2 Amgen Inc., Thousand Oaks, CA USA

**Keywords:** Chemotherapy-induced thrombocytopenia, Myelosuppressive chemotherapy, Bleeding, Thrombopoietin-receptor agonist

## Abstract

**Background:**

Chemotherapy-induced thrombocytopenia (CIT) is a potentially serious complication that can lead to chemotherapy dose delays, dose reductions, or discontinuation, and increases the risk of serious bleeding events. The objectives of this study were to characterize the incidence, clinical consequences, and economic costs of CIT in current US clinical practice.

**Methods:**

A retrospective cohort design and data from two US private healthcare claims repositories (01/2010–12/2016) were employed. Study population comprised adults who received selected myelosuppressive chemotherapy regimens for solid tumors or non-Hodgkin’s lymphoma. CIT was identified based on: diagnosis code for thrombocytopenia or bleeding; procedure code for platelet transfusion or bleeding control; or drug code for thrombopoietin-receptor agonist. Incidence of CIT was evaluated during the chemotherapy course (max. no. cycles = 8), and associated consequences and costs (2016US$) were evaluated during the cycle of the CIT episode.

**Results:**

Among 215,508 cancer chemotherapy patients, CIT incidence during the course (mean no. cycles = 4.6) was 9.7% (95% CI: 9.6–9.8), and ranged from 6.1% (5.9–6.3) for regimens containing cyclophosphamide to 13.5% (12.7–14.3) for regimens containing gemcitabine; among all patients, incidence was 2.7% (2.6–2.8) in cycle 1, 2.7% (2.6–2.8) in cycle 2, and 2.9% (2.9–3.0) in cycles thereafter. One-third of CIT episodes were managed in hospital, and for the subset of patients hospitalized with a first-listed diagnosis of CIT, mean length of stay was 4.6 (4.4–5.0) days and mean cost of inpatient care was $36,448 (32,332-41,331). Across cycles with CIT, mean cost of CIT-related care was $2179 (2029-2329), comprising $1024 (881–1167) for inpatient care and $1153 (1119-1187) for outpatient care.

**Conclusions:**

In this retrospective evaluation of cancer chemotherapy patients, CIT incidence was high, especially among patients receiving gemcitabine-based regimens, and the costs of CIT-related care were substantial. Accordingly, interventions aimed at identifying and targeting high-risk patients for preventative measures may yield substantial clinical and economic benefits.

**Electronic supplementary material:**

The online version of this article (10.1186/s12885-019-5354-5) contains supplementary material, which is available to authorized users.

## Background

Thrombocytopenia, an abnormally low blood platelet count, is a potentially serious and costly complication of myelosuppressive chemotherapy [1, 2]. Chemotherapy-induced thrombocytopenia (CIT) can complicate surgical procedures and can lead to chemotherapy dose delays, dose reductions, or discontinuation, which may result in suboptimal patient outcomes, and CIT increases the likelihood of serious bleeding events, which may result in hospitalization [2–7]. Currently, there are no drugs approved by the United States (US) Food and Drug Administration for the treatment of CIT, and thus options consist of reducing the intensity of or eliminating the offending treatment, platelet transfusions, or pharmacotherapy, although such options are not without risks and costs [1, 3, 4, 8].

Notwithstanding the potential implications of CIT, little is known about the incidence and consequences of this condition in current US clinical practice. To date, only two studies have evaluated the incidence of CIT using US data sources, and the data employed in these studies are now more than a decade old [2, 4]. Moreover, the study by Hitron et al. was based on a small sample of cancer patients receiving high-risk chemotherapy regimens from a single center, while that by Wu and colleagues was based on an electronic medical records database that did not include important information on characteristics of the study population. In addition, only one study has reported the results of a formal evaluation of the cost of CIT, and this study was based on a sample of 75 patients receiving chemotherapy over 20 years ago [5].

Since the incidence, severity, and duration of CIT varies across patient populations and chemotherapy regimens, and because the treatment of cancer has changed markedly over the past two decades, available evidence may not be reflective of current US clinical practice. The current study was therefore undertaken to evaluate the incidence and consequences of CIT among patients receiving selected myelosuppressive chemotherapy regimens for solid tumors or non-Hodgkin’s lymphoma (NHL) using data from two large healthcare claims repositories. The findings of this research were presented, in part, at the American Society of Clinical Oncology (ASCO) Annual Meeting, June 1–5, 2018 [9].

## Methods

### Study design and data sources

This study employed a retrospective cohort design and data from two large healthcare claims repositories spanning the period from January 1, 2010 through December 31, 2016. A detailed description of study methods—design, data source, and operational algorithms for selecting patients as well as identifying all other study variables (including corresponding diagnosis, procedure, and drug codes)—is set forth in Online Additional file 1.

Patient-level claims data from the two repositories were pooled to increase the precision and generalizability of study findings. The two study repositories―Truven Health Analytics MarketScan® Commercial Claims and Encounters and Medicare Supplemental and Coordination of Benefits Databases (“MarketScan Database”), and IQVIA Real-World Data Adjudicated Claims PharMetrics Plus Database (“PharMetrics Database”)―comprise medical (i.e., facility and professional service) claims and outpatient pharmacy claims from a large number of participating private US health plans. The study databases were de-identified prior to their release to study investigators, and thus their use for health services research was fully compliant with the Health Insurance Portability and Accountability Act of 1996 (HIPAA) Privacy Rule and federal guidance on Public Welfare and the Protection of Human Subjects (45 CFR 46 §46.101).

### Source and study populations

The source population comprised patients aged ≥18 years who, between January 1, 2011 and December 31, 2015, initiated ≥1 course of myelosuppressive chemotherapy for a single primary solid tumor or NHL. For each patient in the source population, the first unique observed course of chemotherapy, and each cycle (up to 8 in total) within the first course, was identified. Patients were excluded from the source population if they had: < 6 months of continuous health benefits prior to chemotherapy initiation (e.g., if they were recently enrolled in a participating health plan, and thus their healthcare claims data were not available prior to this period); evidence of stem cell therapy or bone marrow transplant during the 12-month period preceding chemotherapy or during the chemotherapy course; evidence of thrombocytopenia during the 12-month period preceding chemotherapy; or evidence of secondary causes of thrombocytopenia during the 12-month period preceding chemotherapy or during the chemotherapy course.

From the source population, all patients who received chemotherapy regimens including ≥1 agent of interest—carboplatin, cisplatin, cyclophosphamide, fluorouracil, gemcitabine, oxaliplatin, and vincristine—were included in the study population, and all such patients were included in ≥1 non-mutually-exclusive subgroup based on receipt of the agents of interest. The agents of interest were selected based on previously published research suggesting that patients receiving chemotherapy regimens including these drugs were at elevated risk of CIT [1, 7, 10].

### Study outcomes

#### Episodes of CIT

Episodes of CIT were ascertained on a cycle-specific basis during the chemotherapy course, from day 7 of each chemotherapy cycle through the end of the cycle, and were identified based on inpatient/outpatient medical claims with a diagnosis code (in any position) for thrombocytopenia (primary, secondary, or unspecified) or bleeding, or a procedure code for bleeding treatment, platelet transfusion, or receipt of a thrombopoietin receptor agonist (TPO-RA). CIT episodes identified in the ambulatory setting that preceded/followed CIT episodes identified in the inpatient setting during the same cycle were considered part of the inpatient episode (for purposes of stratifying CIT episodes by care setting).

#### Clinical and economic consequences of CIT

For each CIT episode, consequences were evaluated within the cycle of occurrence and included hospital admissions with a first-listed diagnosis code for thrombocytopenia or bleeding; ambulatory encounters with a diagnosis code for thrombocytopenia or bleeding, or a procedure code for selected transfusions, laboratory tests, control of bleeding, or CIT-related medications (i.e., glucocorticosteroids, immunoglobulin, and TPO-RAs); or outpatient pharmacotherapy with CIT-related medications. For CIT-related hospital admissions, consequences were characterized in terms of hospital length of stay, hospital mortality, diagnoses, and cost per admission, as well as in terms of the number of hospitalizations, number of hospital days, and hospital costs per patient-episode. For CIT-related ambulatory encounters, consequences were characterized in terms of care setting, diagnoses, procedures, outpatient pharmacotherapy, and cost per encounter, as well as in terms of the number of ambulatory encounters and ambulatory costs per patient-episode. Numbers and costs of outpatient CIT-related medications per patient-episode, and total cost per patient-episode, were also tallied. Costs were expressed in 2016US$, and were based on amounts paid by health plans and patients for services rendered by providers.

### Patient characteristics

Characteristics of the study population included age; sex; chronic comorbidities; nutritional status; history of other conditions/events prior to chemotherapy; measures of health status and physical function. All baseline characteristics were assessed during the 12-month period preceding chemotherapy initiation, except for recent surgery, which was evaluated during the 90-day period prior to chemotherapy.

### Statistical analyses

Crude risk of CIT—overall and by care setting—was summarized for all patients and for subgroups defined therein based on the agent of interest using incidence proportions and corresponding 95% confidence intervals (CIs); the latter were generated using the Wilson score interval method. CIT-related admissions, ambulatory encounters, and costs were summarized using means, frequencies, and corresponding 95% CIs, which were calculated using techniques of non-parametric bootstrapping. Since some of our measures of CIT-related care (e.g., red blood cell transfusions, labs, and pharmacotherapy) may capture services provided for other reasons (i.e., other than CIT), measures were also evaluated during cycles in which CIT episodes did not occur among our study population, for purposes of comparison. All analyses were also conducted within subgroups defined on cancer type as well as cancer type and chemotherapy agent.

## Results

The two study databases included a total of 643,676 patients aged ≥18 years who received myelosuppressive chemotherapy between January 1, 2011 and December 31, 2015 (Table 1). Among these patients, 332,512 (52%) met all remaining inclusion/exclusion criteria and qualified for inclusion in the source population. From the source population, 215,508 patients also received chemotherapy regimens including an agent of interest—mostly commonly, cyclophosphamide (39%) and carboplatin (28%)—and thus qualified for inclusion in the study population. Mean (SD) age of study population was 57.3 (11.3) years, and 70% were female (Table 2). Common comorbidities included cardiovascular disease (16%), lung disease (16%), and diabetes (15%), and a large percentage of patients had a history of infection (61%) or anemia (21%). Characteristics of patients in subgroups defined in cancer type as well as cancer type and chemotherapy agent (Online Additional file 2: Table S1a-b) and characteristics of chemotherapy regimens and supportive care (Online Additional file 2: Table S2) are reported in Online Additional file 2.

**Table 1:** Selection of source and study populations

	Total Population
Source Population	
Patients aged ≥18 years with evidence of new myelosuppressive chemotherapy course from 1/2011 to 12/2015	643,676
plus ≥6 months continuous health benefits prior to chemotherapy course	507,181
plus evidence of single primary solid tumor or NHL at the time of chemotherapy initiation	359,727
plus no evidence of stem cell/bone marrow transplant prior to or during chemotherapy	359,429
plus no evidence of thrombocytopenia prior to chemotherapy initiation	352,571
plus no evidence of causes of secondary thrombocytopenia prior to or during the chemotherapy course	332,512
Study Population	
Receipt of chemotherapy regimen including one of the following drugs of interest	215,508
Carboplatin	59,381
Cisplatin	18,380
Cyclophosphamide	67,530
Fluorouracil	10,408
Gemcitabine	3704
Oxaliplatin	5867
Vincristine	766
≥ 2 Drugs of Interest	49,472
Carboplatin+Fluorouracil	343
Carboplatin+Gemcitabine	1154
Cisplatin+Cyclophosphamide	145
Cisplatin+Fluorouracil	2462
Cisplatin+Gemcitabine	1318
Cyclophosphamide+Fluorouracil	2117
Cyclophosphamide+Vincristine	13,812
Fluorouracil+Oxaliplatin	27,566
Gemcitabine+Oxaliplatin	363
Other Regimens	192

**Table 2:** Characteristics of patients in study population, by chemotherapy agent^a^

	All Agents	Carboplatin	Cisplatin	Cyclophosphamide	Fluorouracil	Gemcitabine	Oxaliplatin	Vincristine
	(*n* = 215,508)	(*n* = 60,966)	(*n* = 22,393)	(*n* = 83,665)	(*n* = 42,970)	(*n* = 6617)	(*n* = 33,822)	(*n* = 14,610)
Patient								
Age (years), mean (SD)	57.3 (11.3)	59.7 (11.3)	57.6 (10.8)	54.7 (10.9)	58.1 (10.8)	63.3 (11.4)	57.6 (10.7)	59.1 (14.0)
Female, %	69.5	72.8	40.1	89.0	46.9	51.2	42.5	42.5
Chronic Comorbidities, %								
Cardiovascular Disease	16.4	21.4	21.4	9.6	18.5	28.4	19.1	20.7
Cardiac Dysrhythmias	8.1	10.0	9.8	5.0	9.7	14.3	10.3	10.4
Cerebrovascular Disease	3.0	4.8	4.2	1.5	2.5	5.1	2.4	3.5
Ischemic Heart Disease	8.4	11.3	11.9	4.3	9.7	15.9	9.7	10.7
Heart Failure	2.6	3.7	2.6	1.4	2.9	5.2	2.9	3.7
Diabetes	14.7	15.8	14.0	11.7	18.0	25.7	18.2	16.5
Liver Disease	7.7	7.0	6.8	3.7	14.4	19.1	16.7	6.0
Lung Disease	15.5	23.9	26.1	9.0	11.1	18.6	10.8	11.3
Osteoarthritis	10.0	11.5	10.4	9.4	8.9	14.4	8.4	12.2
Renal Disease	3.7	4.6	2.9	2.4	4.6	10.8	4.5	6.4
Nutritional Status								
Malnutrition	2.3	2.3	2.8	0.5	4.8	6.2	5.3	2.1
Obesity	9.1	9.3	7.4	8.3	10.6	8.6	12.1	8.2
History of Other Conditions/Events Prior to Chemotherapy Course, %								
Anemia	20.9	19.9	18.0	12.4	36.2	30.9	41.6	25.9
Neutropenia	2.3	2.3	1.9	3.0	1.2	3.3	1.1	8.6
Infection	60.7	59.8	61.9	60.9	61.8	61.5	63.1	61.2
History of Chemotherapy	4.8	5.0	3.5	5.1	3.4	10.7	2.9	19.0
History of Hospitalization for Any Reason	45.2	48.4	46.8	28.3	65.1	59.4	77.1	39.3
History of Radiation Therapy	16.2	19.0	44.4	4.1	21.6	15.1	13.3	3.9
Recent Surgery	35.2	33.6	26.8	38.0	36.0	30.0	40.6	23.1
Proxies for Health Status								
Hospice, %	0.4	0.5	0.2	0.3	0.5	0.6	0.5	0.4
SNF, %	1.5	2.2	1.2	0.6	1.8	3.7	1.8	2.1
Pre-Chemotherapy Expenditures ($)								
Mean (SD)	48,669 (47,526)	48,179 (50,374)	50,328 (48,500)	40,918 (33,823)	57,176 (53,867)	65,571 (70,875)	62,275 (55,302)	47,412 (49,110)
Proxies for Physical Function, %								
Use of Hospital Bed	0.5	0.8	0.5	0.2	0.6	1.0	0.6	0.4
Use of Supplemental Oxygen	4.4	7.7	7.9	1.8	2.9	6.8	2.7	3.3
Use of Walking Aid	2.5	3.3	2.6	1.4	2.8	5.0	2.9	3.1
Use of Wheelchair	0.8	1.3	0.7	0.4	0.6	1.8	0.6	1.1

^a^Agent-specific subgroups are not mutually exclusive

Incidence of CIT during the chemotherapy course was 9.7% (95% CI: 9.6–9.8) among all patients in the study population, and ranged from 6.1% (5.9–6.3) among those receiving a cyclophosphamide-based regimen to 13.5% (12.7–14.3) among patients receiving a gemcitabine-based regimen; CIT incidence among patients receiving regimens including carboplatin was 13.2% (12.9–13.5) (Table 3). Incidence of CIT among all patients was 2.7% (2.6–2.8) in cycle 1, 2.7% (2.6–2.8) in cycle 2, and 2.9% (2.9–3.0) in subsequent cycles. Approximately one-third of CIT episodes were managed in hospital.

**Table 3:** Incidence of chemotherapy-induced thrombocytopenia, by chemotherapy agent^a^

	Incidence Proportion (95% CI)							
	All Agents	Carboplatin	Cisplatin	Cyclophosphamide	Fluorouracil	Gemcitabine	Oxaliplatin	Vincristine
Evidence of Primary, Secondary, Unspecified Thrombocytopenia and/or Bleeding Events, Receipt of TPOs, or Platelets During Chemotherapy								
Course	(*n* = 215,508)	(*n* = 60,966)	(*n* = 22,393)	(*n* = 83,665)	(*n* = 42,970)	(*n* = 6617)	(*n* = 33,822)	(*n* = 14,610)
Overall	9.7 (9.6–9.8)	13.2 (12.9–13.5)	9.9 (9.5–10.3)	6.1 (5.9–6.3)	10.9 (10.6–11.2)	13.5 (12.7–14.3)	11.4 (11.1–11.8)	9.6 (9.1–10.1)
Inpatient	3.0 (2.9–3.1)	4.2 (4.1–4.4)	4.3 (4.1–4.6)	1.6 (1.5–1.7)	3.0 (2.9–3.2)	5.5 (4.9–6.0)	2.9 (2.7–3.1)	3.5 (3.2–3.8)
Outpatient	6.7 (6.6–6.8)	9.0 (8.7–9.2)	5.6 (5.3–5.9)	4.5 (4.3–4.6)	7.9 (7.6–8.1)	8.0 (7.4–8.7)	8.5 (8.2–8.8)	6.1 (5.7–6.5)
All Cycles	(*n* = 988,195)	(*n* = 251,611)	(*n* = 67,304)	(*n* = 435,897)	(*n* = 210,734)	(n = 22,310)	(*n* = 179,105)	(*n* = 57,387)
Overall	2.8 (2.8–2.9)	4.2 (4.2–4.3)	4.1 (3.9–4.2)	1.5 (1.5–1.5)	3.2 (3.1–3.3)	5.2 (4.9–5.5)	3.3 (3.2–3.4)	3.2 (3.1–3.4)
Inpatient	0.7 (0.7–0.7)	1.1 (1.0–1.1)	1.5 (1.4–1.6)	0.3 (0.3–0.3)	0.6 (0.6–0.7)	1.7 (1.5–1.9)	0.6 (0.5–0.6)	0.9 (0.9–1.0)
Outpatient	2.1 (2.1–2.2)	3.2 (3.1–3.2)	2.6 (2.4–2.7)	1.2 (1.1–1.2)	2.6 (2.5–2.6)	3.5 (3.2–3.7)	2.7 (2.7–2.8)	2.3 (2.2–2.4)
Cycle 1	(*n* = 215,508)	(*n* = 60,966)	(*n* = 22,393)	(*n* = 83,665)	(*n* = 42,970)	(*n* = 6617)	(*n* = 33,822)	(*n* = 14,610)
Overall	2.7 (2.6–2.8)	3.9 (3.7–4.0)	3.7 (3.5–4.0)	1.7 (1.6–1.8)	2.0 (1.9–2.2)	7.0 (6.4–7.6)	1.5 (1.4–1.7)	4.1 (3.8–4.4)
Inpatient	1.0 (1.0–1.0)	1.4 (1.4–1.5)	1.7 (1.5–1.9)	0.6 (0.5–0.6)	0.7 (0.6–0.8)	3.0 (2.6–3.5)	0.5 (0.4–0.6)	1.5 (1.3–1.7)
Outpatient	1.7 (1.7–1.8)	2.4 (2.3–2.6)	2.0 (1.9–2.2)	1.2 (1.1–1.2)	1.3 (1.2–1.4)	4.0 (3.5–4.4)	1.0 (0.9–1.1)	2.6 (2.3–2.8)
Cycle 2	(*n* = 186,607)	(*n* = 51,796)	(*n* = 17,777)	(*n* = 76,661)	(*n* = 36,785)	(*n* = 4255)	(*n* = 29,562)	(*n* = 11,552)
Overall	2.7 (2.6–2.8)	3.7 (3.6–3.9)	3.9 (3.6–4.2)	1.4 (1.4–1.5)	2.9 (2.8–3.1)	5.1 (4.4–5.7)	2.5 (2.3–2.7)	3.0 (2.7–3.3)
Inpatient	0.8 (0.7–0.8)	1.0 (0.9–1.1)	1.4 (1.2–1.6)	0.3 (0.2–0.3)	1.0 (0.9–1.1)	1.4 (1.1–1.8)	0.7 (0.6–0.8)	0.8 (0.7–1.0)
Outpatient	1.9 (1.9–2.0)	2.7 (2.6–2.8)	2.5 (2.3–2.7)	1.2 (1.1–1.3)	2.0 (1.8–2.1)	3.7 (3.1–4.2)	1.8 (1.6–1.9)	2.1 (1.9–2.4)
Cycle 3+	(*n* = 586,080)	(*n* = 138,849)	(*n* = 27,134)	(*n* = 275,571)	(*n* = 130,979)	(*n* = 11,438)	(*n* = 115,721)	(*n* = 31,225)
Overall	2.9 (2.9–3.0)	4.6 (4.5–4.7)	4.4 (4.2–4.7)	1.4 (1.4–1.5)	3.7 (3.6–3.8)	4.1 (3.8–4.5)	4.0 (3.9–4.1)	2.9 (2.7–3.1)
Inpatient	0.5 (0.5–0.6)	0.9 (0.9–1.0)	1.4 (1.2–1.5)	0.3 (0.2–0.3)	0.5 (0.5–0.5)	1.0 (0.8–1.2)	0.5 (0.5–0.6)	0.7 (0.6–0.8)
Outpatient	2.4 (2.3–2.4)	3.7 (3.6–3.8)	3.0 (2.8–3.3)	1.2 (1.1–1.2)	3.1 (3.1–3.2)	3.1 (2.8–3.4)	3.5 (3.4–3.6)	2.2 (2.0–2.3)

^a^Only first eight cycles were considered in analyses described herein; agent-specific subgroups are not mutually exclusive

For hospital admissions with a principal diagnosis of CIT, mean length of stay was 4.6 (4.4–5.0) days, hospital mortality was 4.7% (2.8–6.6), and mean cost of inpatient care was $36,448 (32,332-41,331) (Table 4). For CIT-related ambulatory encounters, most of which occurred in a physician’s office (45%) or hospital outpatient department (41%), mean cost per encounter was $123 (121–125) for the former and $504 (486–524) for the latter. Across cycles with CIT, irrespective of care setting, mean number of CIT-related admissions was 0.03 (0.03–0.03) per patient-episode, and the mean number of CIT-related ambulatory encounters was 3.7 (3.7–3.8) per patient-episode; the total cost of CIT-related care was $2179 (2029-2329) per patient-episode, comprising $1024 (881–1167) for inpatient care and $1153 (1119-1187) for outpatient care. For purposes of comparison, across cycles without CIT, the cost of selected transfusions, labs, and pharmacotherapy (i.e., services that may have been provided for reasons other than CIT) totaled $271 (265–278) per patient-cycle. Incidence and burden of CIT within subgroups defined on cancer type as well as cancer type and chemotherapy agent are reported in Online Additional file 2 (Online Additional file 2: Table S3a-4b).

**Table 4:** Treatment and consequences of CIT requiring inpatient or outpatient care

	All Agents
	(*n* = 27,913)
Number of CIT-Related Hospital Admissions, mean per patient-episode (95% CI)	0.03 (0.03–0.03)
Characteristics of Admissions (i.e., per admission)	
LOS, days, mean (95% CI)	4.6 (4.4–5.0)
Mortality^a^, % (95% CI)	4.7 (2.8–6.6)
Diagnoses, % (95% CI)	
CIT Only	7.7 (5.7–9.6)
Bleeding Only	85.2 (82.5–87.6)
CIT and Bleeding	7.1 (5.4–9.1)
Cost ($), mean per admission (95% CI)	36,448 (32,332 - 41,331)
Number of Hospital Days, mean per patient-episode (95% CI)	0.13 (0.12–0.14)
Cost of Admissions ($), mean per patient-episode (95% CI)	1024 (881–1167)
Number of CIT-Related Ambulatory Encounters, mean per patient-episode (95% CI)	3.7 (3.7–3.8)
Characteristics of Outpatient Encounters (i.e., per encounter)	
Setting of Care, % (95% CI)	
Physician Office	44.6 (44.3–44.9)
Emergency Department	2.2 (2.1–2.3)
Hospital Outpatient	40.7 (40.4–41.0)
Labs	4.5 (4.4–4.6)
Other	8.0 (7.8–8.1)
Diagnoses, % (95% CI)	
CIT Only	16.2 (16.0–16.4)
Bleeding Only	15.8 (15.6–16.0)
CIT and Bleeding	0.4 (0.4–0.5)
None of the Above	67.5 (67.2–67.8)
Procedures, % (95% CI)	
Blood Product Transfusion	
Platelet	2.0 (1.9–2.1)
Red Blood	3.1 (3.0–3.2)
Stem Cell	0.2 (0.2–0.2)
Whole Blood	0.0 (0.0–0.0)
Other	0.4 (0.3–0.4)
Coagulation Factor Transfusion	0.0 (0.0–0.0)
Control of Bleeding	0.7 (0.6–0.7)
Labs	64.1 (63.8–64.4)
Pharmacotherapy, % (95% CI)	
Glucocorticosteroid	28.3 (28.0–28.6)
Immunoglobulin	0.0 (0.0–0.0)
Thrombopoietin Receptor Agonists	
Eltrombopag	---
Romiplostim	0.0 (0.0–0.0)
Cost ($), mean per encounter (95% CI)	
Physician Office	123 (121–125)
Emergency Department	839 (779–918)
Hospital Outpatient	504 (486–524)
Labs	11 (11–11)
Other	359 (337–383)
Cost of Ambulatory Encounters ($), mean per patient-episode (95% CI)	1153 (1119 - 1187)
Characteristics of Outpatient Pharmacotherapy	
Number of Filled Prescriptions, mean per patient-episode (95% CI)	0.2 (0.2–0.2)
Glucocorticosteroid, % (95% CI)	99.9 (99.8–100.0)
Immunoglobulin, % (95% CI)	0.1 (0.0–0.1)
Thrombopoietin Receptor Agonists, % (95% CI)	
Eltrombopag	0.0 (0.0–0.1)
Romiplostim	0
Cost ($), mean per prescription (95% CI)	
Glucocorticosteroid	10 (10–11)
Immunoglobulin	7302 (6735 - 7869)
Thrombopoietin Receptor Agonists	
Eltrombopag	5640 (5038 - 6242)
Romiplostim	0
Cost of Prescriptions ($), mean per patient-episode (95% CI)	3 (2–4)
Total Cost per Cycle ($), mean per patient-episode	2179 (2029 - 2329)

^a^Hospital mortality was based on data from the MarketScan Database only

## Discussion

Using a retrospective cohort design and data from two large healthcare claims repositories, we undertook an evaluation to better understand the current epidemiology and burden of CIT among persons receiving selected myelosuppressive chemotherapy regimens for solid tumors or NHL in US clinical practice. Our findings indicate that the incidence of CIT during the chemotherapy course is high (~ 10%), especially among patients receiving gemcitabine- or carboplatin-based chemotherapy regimens. Because episodes of CIT were identified based on evidence of thrombocytopenia or bleeding documented during healthcare encounters (and not via laboratory results), we believe our algorithm disproportionately captures more severe cases of disease and thus our estimates may not be reflective of the overall incidence of CIT including all cases, irrespective of disease severity.

Our estimates of CIT incidence are, not surprisingly, generally lower than those previously published, presumably due in large part to differences in case-ascertainment algorithms. In the study by Wu and colleagues, which utilized electronic medical records for patients (*n* = 47,159) treated in US oncology clinics between 2000 and 2007, CIT incidence—defined as a platelet count < 150 × 10^9^ /L—among all patients was estimated to be 41.4%, ranging from 21.2% among patients receiving taxane-based regimens to 64.2% among patients who received gemcitabine-based regimens [2]. However, when employing the same threshold for CIT as employed in the smaller study published by Hitron et al. (i.e., platelet count < 75 × 10^9^ /L, which along with other evidence, was considered to be clinically significant), overall incidence based on data from Wu et al. was calculated to be 13.8%. This estimate based on data from Wu et al. is comparable to CIT incidence reported by Hitron and colleagues (11.2%), CIT incidence reported in an ex-US study (11.9%, with CIT defined as platelet count < 75 × 10^9^ /L), as well as that reported in the current study [4, 11].

Our findings also indicate that the economic costs of CIT-related care are substantial, averaging $2179 per episode, or $1908 more than cycles without CIT. Notwithstanding differences in study design, study populations, and CIT definitions, our estimate of the cost of CIT-related care is comparable to that reported previously by Elting and colleagues [5]. In their 2003 analysis of 75 patients with a solid tumor or lymphoma who developed thrombocytopenia during chemotherapy treatment, mean incremental cost of CIT was estimated to be $1037 in 1999US$, or $2011 in 2016US$. Among our 27,913 cycles with evidence of CIT, 75% (*n* = 20,920) had evidence of bleeding, and CIT-related costs in these cycles were substantially higher than in the 25% (*n* = 6993) of cycles without evidence of bleeding. In cycles with bleeding, mean costs of CIT-related care (per patient-episode) totaled $2632, including $1349 for CIT-related hospital admissions and $1281 for CIT-related ambulatory care; corresponding values for cycles without bleeding were $823 (total), $51 (hospital), and $770 (ambulatory).

We note several potential biases vis-à-vis patient (treatment) selection and outcome assessment that may impact the findings of this study as well as important limitations. The accuracy of the algorithm for identifying patients receiving chemotherapy for solid tumors and NHL is unknown, as it has not been validated. However, similar algorithms for identifying cancer chemotherapy patients in healthcare claims databases have been employed in several previously published studies [12–14]. Because a diagnosis code for CIT does not exist, and the study database does not include results of laboratory tests (i.e., platelet counts), an algorithm based on diagnosis codes for thrombocytopenia and bleeding, and procedure codes for bleeding treatment, platelet transfusion, and TPO-RA therapy, was employed to identify CIT. While the accuracy of this algorithm is unknown, we suspect—as noted above—that patients with less severe CIT may be disproportionately under-represented, and thus the estimated cost of CIT-related care must be interpreted accordingly. We also note that because thrombocytopenia, neutropenia, and anemia are all known hematological toxicities of myelosuppressive chemotherapy, the reported incremental cost of CIT may be, to some extent, confounded by the presence of anemia and/or neutropenia, and the impact of such bias is unknown. Algorithms and variables employed to identify chronic comorbidities and acute illnesses have not been validated, and their accuracy is unknown; also, some patients may be misclassified in terms of their characteristics because healthcare claims are available only during the study period. Data on hospital discharge disposition were available only in the MarketScan Database, and thus were assumed to be generalizable to the overall population of patients hospitalized for CIT. Health plans contributing claims data to the two study databases are different. However, patients may be insured by more than one health plan at the same time, and thus their healthcare claims may be included in both of the study databases; the magnitude of overlap is believed to be negligible. Finally, caution is warranted in generalizing results beyond this evaluation as the study population was disproportionately represented by non-elderly patients with commercial healthcare coverage.

## Conclusions

In conclusion, in this retrospective evaluation of cancer chemotherapy patients, CIT incidence was high, especially among patients receiving gemcitabine-based regimens, and the costs of CIT-related care were substantial. Accordingly, interventions aimed at identifying and targeting patients at high risk of CIT for preventative measures may yield substantial clinical and economic benefits.

## Additional file

Additional file 1: Risk and Consequences of CIT v5, Study Methods and Appendices, description of study methods and operational algorithms/codes used to define study variables. (DOC 312 kb)

Additional file 2: Risk and Consequences of CIT v2.1, Supplemental Findings, description of results from analyses within subgroups defined on cancer type and chemotherapy agent. (XLSX 327 kb)

## Abbreviations

ASCO: American Society of Clinical Oncology
CI: Confidence interval
CIT: Chemotherapy-induced thrombocytopenia
HIPAA: Health Insurance Portability and Accountability Act
MarketScan Database: Truven Health Analytics MarketScan® Commercial Claims and Encounters and Medicare Supplemental and Coordination of Benefits Databases
NHL: Non-Hodgkin’s lymphoma
PAI: Policy Analysis Inc.
PharMetrics Database: IQVIA Real-World Data Adjudicated Claims PharMetrics Plus Database
TPO-RA: Thrombopoietin receptor agonist
US: United States
