# Supplementary material for: Risk and consequences of chemotherapy-induced thrombocytopenia in US clinical practice
Source: BMC Cancer. 2019 Feb 14;19:151. doi: 10.1186/s12885-019-5354-5 (PMC6376753; doi:10.1186/s12885-019-5354-5)
Supplement: Supplementary file 1 — Risk and Consequences of CIT v5, Study Methods and Appendices, description of study methods and operational algorithms/codes used to define study variables. (DOC 312 kb) [file 12885_2019_5354_MOESM1_ESM.doc]

ONLINE ADDITIONAL FILE 1:

**STUDY METHODS**

# METHODS

## Study Design

This study employed a retrospective cohort design and data from two large US private healthcare claims repositories. Patient level claims from the two repositories were pooled for analyses.

**
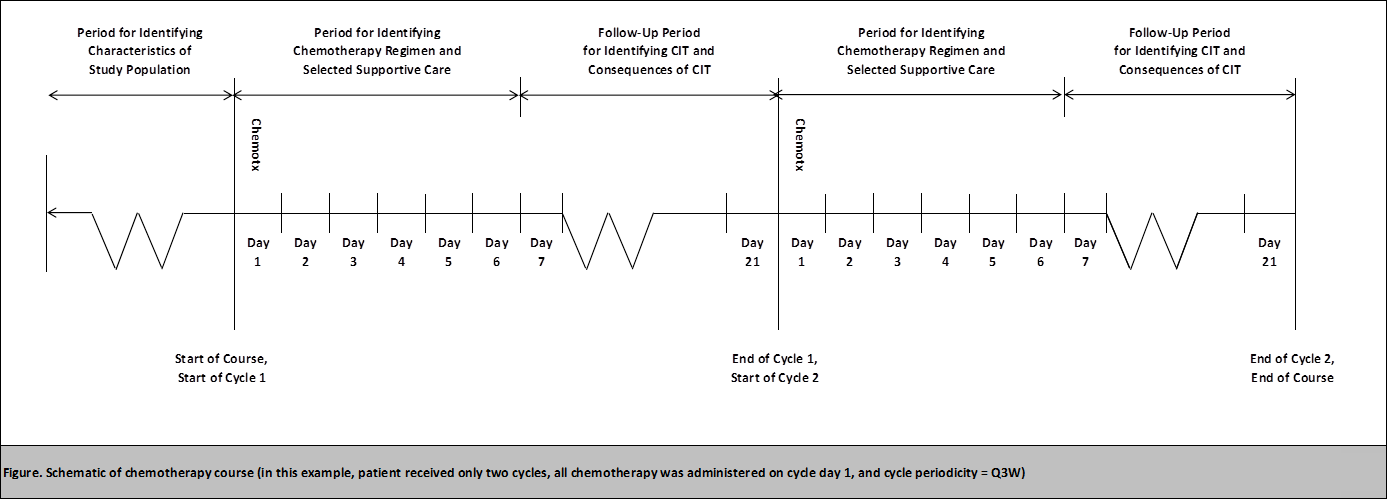
**

## Data Source Population

The data source population comprised (principally) persons who have private employer-sponsored healthcare coverage, along with their spouses and dependents. Elderly persons who are Medicare-eligible and have elected to enroll in the Medicare Advantage Program or in a Medicare supplemental plan—and thus receive their healthcare coverage, in part or in full, through a private health plan—also were included in the data source population.

## Data Source(s)

The two study repositories―Truven Health Analytics MarketScan® Commercial Claims and Encounters and Medicare Supplemental and Coordination of Benefits Databases (“MarketScan Database”); IQVIA Real-World Data Adjudicated Claims PharMetrics Plus Database (“PharMetrics Database”)―comprise medical (i.e., facility and professional service) and outpatient pharmacy claims from a large number of participating health plans and spanned the period from January 1, 2010 through December 31, 2016.

The MarketScan Database includes information (primarily) from employer-sponsored plans throughout the US that provide health benefits to >15 million persons annually, including employees, their spouses, and their dependents, 10% of whom are aged ≥65 years. The PharMetrics Database includes information from >75 US private health plans providing healthcare coverage to a geographically-diverse population of >15 million persons annually; 4% of plan members are ≥65 years of age. The MarketScan and PharMetrics Databases include information from unique health plans/organizations and have been previously integrated for studies conducted by study authors.

Data available from each facility and professional-service claim include dates and places of service, diagnoses, procedures performed/services rendered, and quantity of services (professional-service claims only); data on discharge disposition is available on inpatient facility claims (MarketScan Database only). Data available for each outpatient pharmacy claim include the drug (class) dispensed, dispensing date, quantity dispensed, and number of days supplied. Medical and pharmacy claims also include amounts paid (i.e., reimbursed) by health plans, as well as by patients, for healthcare services rendered. Selected demographic and eligibility information (including age, sex, geographic region of residence, dates of plan eligibility) is available for all health plan enrollees in the databases. All data can be arrayed to provide a detailed chronology of medical and pharmacy services used by each plan member over time.

## Patient Eligibility

## The source population comprised all patients aged ≥18 years who, from January 1, 2011 through December 31, 2015, received ≥1 course of myelosuppressive chemotherapy for a single solid tumor or NHL (defined below), and met minimum health benefit eligibility criteria requiring at least 6 months of continuous coverage. Patients with evidence of stem cell or bone marrow transplant at any time, evidence of thrombocytopenia prior to chemotherapy initiation, or evidence of causes of secondary thrombocytopenia (e.g., viral hepatitis B, acute/chronic hepatitis C, HIV, or pancytopenia) prior to or during the chemotherapy course were excluded from the source population. For each patient in the source population, the first observed qualifying course of chemotherapy, and each cycle of chemotherapy within that course, was identified (as defined in Section 1.4.1). From the source population, all patients who received chemotherapy regimens that contained ≥1 agent of interest, and that was administered on a biweekly, triweekly, or monthly basis, were selected for inclusion in the study population. For patients who received more than one unique course of chemotherapy—and met all eligibility criteria—only the first qualifying course was considered in analyses.

### Inclusion/Exclusion Criteria

Patients who satisfied the following criteria were selected for inclusion in the source population:

- Aged ≥18 years with evidence of receipt of ≥1 course of myelosuppressive chemotherapy between January 1, 2011 and December 31, 2015;
- ≥6 months of continuous health benefits prior to chemotherapy course;
- Evidence of a single solid tumor, or NHL at the time of chemotherapy initiation;
- No evidence of stem cell or bone marrow transplant prior to or during chemotherapy;
- No evidence of thrombocytopenia prior to chemotherapy initiation; or
- No evidence of causes of secondary thrombocytopenia (e.g., viral hepatitis B, acute/chronic hepatitis C, HIV, or pancytopenia) prior to or during the chemotherapy course.

From the source population, all those patients who received chemotherapy with a regimen that included ≥1 of the following chemotherapy drugs of interest, and that was administered on a biweekly, triweekly, or monthly basis, were selected for inclusion in the study population:

- Carboplatin;
- Cisplatin;
- Cyclophosphamide;
- Fluorouracil;
- Gemcitabine;
- Oxaliplatin; or
- Vincristine.

Patients selected for inclusion in the study population were assigned to ≥1 non-mutually-exclusive subgroup based on receipt of the agents listed above.

**Primary and Secondary Cancers.** Presence of solid tumors and NHL were identified based on ≥2 encounters (≥7 days apart) with a qualifying ICD-9-CM/ICD-10-CM diagnosis code (Appendix A) during the period beginning 30 days prior to the date of chemotherapy initiation and ending 30 days thereafter. Presence of metastasis was identified on the basis of ≥1 ICD-9-CM/ICD-10-CM diagnosis code on inpatient claims or ≥2 diagnosis codes on outpatient claims (excluding those for laboratory services) on different days during the 1-year period prior to chemotherapy initiation (Appendix B).

**Chemotherapy Courses and Cycles.** For each cancer chemotherapy patient, each unique cycle within the first observed course of chemotherapy was identified. The first chemotherapy cycle was defined as beginning with the date of chemotherapy initiation and ending with the first service date for the next administration of chemotherapy (as evidenced by an encounter with a corresponding HCPCS or ICD-9-CM/ICD-10-CM code) occurring at least 7 days—but no more than 89 days—after the date of initiation of chemotherapy. If a second chemotherapy cycle did not commence prior to day 90, or if there was an unplanned change in the chemotherapy regimen (i.e., based on expert opinion regarding agents received in the first cycle versus subsequent cycles and cycle periodicity [i.e., cycle duration was reduced]), both the first cycle of chemotherapy and the course of chemotherapy were considered to have been completed 35 days following the beginning of the cycle or on the date of unplanned change in the regimen, whichever occurred first. The second and all subsequent cycles of chemotherapy—up to eight in total—during the first qualifying course were similarly defined.

Only myelosuppressive chemotherapy agents (Appendix C) were considered in characterizing courses and cycles therein. Chemotherapy courses were characterized based on observed patterns of administration using information captured in claims—including corresponding procedure/revenue codes (HCPCS, ICD-9-CM/ICD-10-CM, and UB-92) and dates of service—as well as clinical expertise regarding the regimens and their patterns of use in clinical practice.

**Chemotherapy Regimens.** Chemotherapy regimens were ascertained based on a review of all HCPCS Level II codes for parenterally administered antineoplastic agents (myelosuppressive and non-myelosuppressive) on claims with service dates within 6 days of the start of the first cycle of chemotherapy. Chemotherapy regimens were characterized based on the agents received as well as cycle periodicity (i.e., weekly [QW], every two weeks [Q2W], every three weeks [Q3W], every four weeks [Q4W], which were based on the observed interval between the first and second cycles). Adjustments to the algorithms that were used to characterize chemotherapy courses, cycles, and regimens commonly used in clinical practice (e.g., regimens including multiple administrations of drugs separated by ≥7 days within a given cycle, sequential regimens [AC🡪T]) were made on an as-needed basis.

## Definitions

### Definitions of Time Periods

#### Study Period

The study period spanned from January 1, 2010 to December 31, 2016.

#### Baseline Period

The baseline period for evaluating characteristics of patients spanned 12 months prior to chemotherapy initiation.

#### Study Follow-up Period

Episodes of CIT were evaluated on a cycle-specific basis, from day 7 of each chemotherapy cycle through the end of the cycle. The treatment and consequences of CIT were evaluated within the cycle of occurrence, from day 7 of each chemotherapy cycle through the end of the cycle. Only the first eight cycles of the chemotherapy course were considered in analyses.

#### Endpoint(s)/Outcomes(s) Assessment

**CIT Episodes.** Episodes of CIT were ascertained on a cycle-specific basis during the chemotherapy course, from day 7 of each chemotherapy cycle through the end of the cycle, and were identified based on a mapped algorithm—utilizing ICD-9-CM/ICD-10-CM diagnosis codes and CPT/HCPCS codes—as follows:

- CIT episodes requiring inpatient care (“Inpatient CIT”), defined as an admission to an acute-care hospital with a principal (i.e., first-listed) or secondary diagnosis code for:
  - Primary thrombocytopenia, unspecified (ICD-9-CM 287.30);
  - Other primary thrombocytopenia (ICD-9-CM 287.39/ICD-10-CM D69.49)
  - Secondary thrombocytopenia (ICD-9-CM 287.4x [excluding 287.41]/ICD-10-CM D69.5x [excluding D69.51]);
  - Unspecified thrombocytopenia (ICD-9-CM 287.5/ICD-10-CM D69.6);
  - Bleeding (Appendix D); or
  - Treatment of bleeding (Appendix E).
- CIT episodes requiring outpatient care only (“Outpatient CIT”), defined as an ambulatory encounter:
  - With a diagnosis code (in any position) for:
    - Primary thrombocytopenia, unspecified (ICD-9-CM 287.30);
    - Other primary thrombocytopenia (ICD-9-CM 287.39/ICD-10-CM D69.49)
    - Secondary thrombocytopenia (ICD-9-CM 287.4x [excluding 287.41]/ICD-10-CM D69.5x [excluding D69.51]);
    - Unspecified thrombocytopenia (ICD-9-CM 287.5/ICD-10-CM D69.6);
    - Bleeding; or
    - Treatment of bleeding.

OR

- - - With a CPT or HCPCS Level II procedure code for (Appendix F):
    - Receipt of TPO-RAs; or
    - Receipt of platelet transfusion.

Outpatient CIT that preceded/followed Inpatient CIT during the same cycle was not considered as a separate episode.

**Clinical and Economic Consequences of CIT.** For each CIT episode, clinical consequences were evaluated within the cycle of occurrence and included hospital admissions with a first-listed diagnosis code for thrombocytopenia or bleeding; ambulatory encounters with a diagnosis code for thrombocytopenia or bleeding, or a procedure code for selected transfusions, laboratory tests, or control of bleeding (Appendix E); or outpatient pharmacotherapy with CIT-related medications (i.e., glucocorticosteroids, immunoglobulin, and TPO-RAs). For CIT-related hospital admissions, consequences were characterized in terms of length of stay, mortality, diagnoses, and cost per admission, as well as in terms of the number of hospitalizations, number of hospital days, and hospital costs per patient-episode (including those with and without CIT). For CIT-related ambulatory encounters, consequences were characterized in terms of care setting, diagnoses, procedures, outpatient pharmacotherapy, and cost per encounter, as well as in terms of the number of ambulatory encounters and ambulatory costs per patient-episode. Numbers and costs of outpatient CIT-related medications per patient-episode, and total cost per patient-episode, were also tallied. Costs were expressed in 2016US$, and were based on amounts paid by health plans and patients for services rendered by providers.

#### Other Study Variables

Characteristics described below represent those considered in published evaluations of CIT and other blood disorders.[^1-3^](#_ENREF_4) Demographic characteristics were available for nearly all study subjects. All other characteristics were defined based on the presence of specific data (e.g., diagnosis codes, procedure codes); the absence of such data was assumed to indicate the absence of the characteristic captured by the variable. While the accuracy of variables capturing healthcare encounters and use of pharmacotherapy (e.g., TPO-RAs) is expected to be high, the accuracy of variables capturing the presence of acute and chronic conditions is undoubtedly less. Characteristics of patients, their cancer, and their treatment included:

- Patient Characteristics: age, sex, chronic comorbidities (cardiovascular disease, cerebrovascular disease, diabetes, liver disease, lung disease, osteoarthritis, renal disease), nutritional status (obesity, malnutrition), proxies for health status (pre-chemotherapy healthcare expenditures, hospice/SNF care), proxies for physical function (use of hospital bed, supplemental oxygen, walking aid, wheelchair), history of blood disorders (anemia, neutropenia, other), history of infection, history of hospitalization;
- Treatment Characteristics: history of chemotherapy, history of radiation therapy, recent surgery, chemotherapy regimen, use of supportive care (CSFs and ESAs [Appendix G]), radiation therapy during chemotherapy course.

Age was assessed as of the first day of the first cycle of chemotherapy during the course. All of other characteristics—unless otherwise noted—were assessed during the 12-month period ending on the day prior to chemotherapy initiation. Recent surgery was assessed during the 90-day pre-chemotherapy period (Appendix H). Chronic comorbidities were identified on the basis of ≥1 diagnosis codes on inpatient claims, ≥2 diagnosis codes on outpatient claims (excluding those for laboratory services) on different days, ≥1 procedure codes, and ≥1 drug codes, as appropriate (Appendix I). Blood disorders and infections were identified on the basis of ≥1 diagnosis codes (on inpatient and/or outpatient claims) and ≥1 drug codes, as appropriate (Appendix J).

ONLINE ADDITIONAL FILE 1:

APPENDICES

Appendix H

*Corresponding ICD-10-CM codes available upon request

ONLINE SUPPLEMENT:

REFERENCES

1. Weycker D, Chandler D, Barron R, et al. Risk of infection among patients with non-metastatic solid tumors or non-Hodgkin's lymphoma receiving myelosuppressive chemotherapy and antimicrobial prophylaxis in US clinical practice. *Journal of oncology pharmacy practice : official publication of the International Society of Oncology Pharmacy Practitioners* 2017;23:33-42.

2. Weycker D, Li X, Edelsberg J, et al. Risk and Consequences of Chemotherapy-Induced Febrile Neutropenia in Patients With Metastatic Solid Tumors. *Journal of oncology practice* 2015;11:47-54.

3. Elting LS, Rubenstein EB, Martin CG, et al. Incidence, cost, and outcomes of bleeding and chemotherapy dose modification among solid tumor patients with chemotherapy-induced thrombocytopenia. *J Clin Oncol* 2001;19:1137-46.
